# Stepping volume and intensity patterns in a multi-ethnic urban Asian population

**DOI:** 10.1186/s12889-018-5457-y

**Published:** 2018-04-23

**Authors:** Jennifer Sumner, Léonie Uijtdewilligen, Anne HY Chu, Sheryl HX Ng, Tiago V. Barreira, Robert Alan Sloan, Rob M. Van Dam, Falk Müller-Riemenschneider

**Affiliations:** 1 Saw Swee Hock School of Public Health, National University of Singapore and National University Health System, Singapore, Singapore; 2 Department of Health Sciences, University of York, York, UK; 3 Syracuse University School of Education, Syracuse, USA; 4 Kagoshima University Graduate School of Medical and Dental Sciences, Kagoshima, Japan; 5 Institute for Social Medicine, Epidemiology and Health Economics, Charite University Medical Centre Berlin, Berlin, Germany

**Keywords:** Physical activity, Steps, Ethnicity, Adult

## Abstract

**Background:**

Accelerometer measured physical activity (PA) studies particularly in non-western populations are lacking. Therefore, this study investigated stepping activity in a multi-ethnic urban Asian population.

**Methods:**

Adult participants from the Singapore Health Study 2 consented to accelerometer activity monitoring for 7-consecutive days. Mean daily step count, peak stepping intensity (i.e. cadence) over 1-min, 30-min and 60-min and time spent in each cadence band: 0 (non-movement), 1–19, 20–39, 40–59, 60–79, 80–99 and ≥ 100 steps/minute (moderate to vigorous PA) were calculated.

**Results:**

A total of 713 participants (42% male, mean age 47.8 years) were included. Overall, the mean daily step count was 7549. Mean daily step count was significantly lower in Indians (7083 adjusted *p* = 0.02) but not Malays 7140 (adjusted *p* = 0.052) compared to Chinese (7745 steps). The proportion of Malays, Indians, and Chinese achieving < 5000 daily steps was 26%, 23% and 14%, respectively (*p* < 0.01). Regardless of ethnicity, approximately half of the recorded time was spent undertaking 0-steps/minute (7.9 h).

**Conclusions:**

Greater promotion of brisk walking is required in light of the low step volume and pace observed in this multi-ethnic Asian population. Ethnic differences in stepping activity were also identified which indicates a need for targeted ethnic specific health promotion interventions.

## Background

Regular physical activity (PA) has repeatedly been shown to be associated with good overall health. International guidelines, such as those formulated by the World Health Organisation (WHO), recommend that adults undertake at least 150-min of moderate intensity aerobic PA/week, which can reduce the risk of developing certain cancers, type 2 diabetes, cardiovascular diseases, falling and mental health issues [1]. Even unstructured PA such as walking and incidental activity can be a major contributor to daily PA [2, 3]. A PA survey of 15 European countries reported approximately 37% of the studied population walked 30 min a day, five times/week [4]. Daily walking goals, such as 10,000 steps, are frequently publicised in health promotion strategies [5, 6] and in pedometer-measured studies 10,000 steps is considered a heuristic marker i.e. practical, for being an ‘active’ adult [7]. Walking activity specifically has also been linked to reductions in cardio-metabolic risk factors [8] and mortality [9], with brisk walking rather than slower paced walking reportedly associated with larger impacts on chronic disease risk [10]. Furthermore, walking is an easy to perform activity even to older adults as it requires no equipment or specialist training and can easily be incorporated into routine life i.e. active transportation to work, as such it is often the focus of public health initiatives [5, 6].

Despite clearly defined guidelines and the ability to accumulate PA from a number of sources, a large proportion of global populations do not reach PA goals and inactivity has contributed to the rise in non-communicable diseases (NCDs) [11]. An estimated 6–10% of NCDs are caused by inactivity and 9% of premature deaths [11]. Comparisons between Western and urban Asian countries have observed lower levels of physical activity in urban Asians [2]. However, much of the research on PA to date is limited to self-reported assessment of PA and there is a need to assess PA using different approaches i.e. measurement with accelerometer [12]. Accelerometer data on PA is also largely limited to Western populations, which may not reflect PA in other ethnic groups. This is concerning given the predisposition of Asians to metabolic disorders such as type 2 diabetes, for which engagement in PA could reduce the risk of such diseases [13].

Measuring steps is a popular way to determine PA, especially considering the increasingly widespread use of consumer PA trackers. However, assessment of stepping volume alone is limited as it does not factor in the intensity of the PA; the focus of PA recommendations, nor does it consider periods of sedentary behaviour. To address this gap, research has recently applied cadence measurement in free living populations i.e. the number of steps taken/minute. Incorporating a measure of cadence into accelerometer measured PA research can then be used to assess whether PA goals (i.e. 150 min/week) are met through stepping based activities [14]. Laboratory work has shown a cadence of ≥100steps/minute to be equivalent to absolute metabolic equivalent (METS) of ≥3 i.e. moderate- to vigorous PA [15]. In addition, cadence measurement can also be used to explore the distribution of PA in greater detail e.g. peak effort, proportion of time at different intensities and variation in intensity across a period of time [16–18]. Research from the US already seems to show limited contributions from stepping towards intensity based PA goals in adults [18], but it is unknown whether the same is true in Asian populations.

To address identified gaps in the literature the aim of this study was to investigate stepping activity in a multi-ethnic urban Asian population using accelerometers and to examine if differences in stepping volume and intensity were apparent between different ethnic groups.

## Methods

Participants of the Singapore Health Study 2 (SHS2) (*n* = 2686) were approached to take part in a further investigation into accelerometer measured PA, which formed the population for this study. Participants of SHS2 were offered to participate in different additional studies as part of the consent procedure (one by one), including this PA study. Those who agreed to participate in the accelerometer measured PA study were subsequently enrolled.

The SHS2, is a cross-sectional representative health survey of participant’s residing in Singapore. A random sample of households were mailed regarding the survey between 2014 and 2015 and followed up through home visits with a trained interviewer. Eligible participants were permanent residents born between 1933 and 1994. Exclusion criteria for participation in the survey were: being pregnant, having a severe mental retardation or mental illness, having had a stroke or injury resulting in speech impairment or being bedridden or wheelchair bound. Information on socio-demographic characteristics and clinical history were captured during a home visit with a researcher and included: age (years), gender (male or female), marital status (married or single: separated/divorced/widowed), educational level (low: no formal qualifications/primary school leaving exam/secondary education, medium: 0-levels/ A-levels, high: diploma/ university degree or equivalent), employment status (working: currently working part or full-time/student/national service or unemployed: home-maker, retired or unemployed), monthly household income (in Singapore dollars), Body Mass Index (BMI) determined from self-reported height and weight, diagnosis of hypertension, diabetes, asthma or arthritis (yes/no), tobacco use (smoker: currently smoking cigarettes), alcohol use (drinker: someone who has consumed alcohol in the last 12 months). Only those of Chinese, Malay and Indian ethnicity, the main ethnic groups in Singapore, were included in the current analysis. Other ethnicities were excluded due to small sample size (*n* = 29).

Participants were provided with an accelerometer (ActiGraph GT3X+, ActiGraph Corp. Pensacola, FL, USA) and instructed to wear the device continuously for 7 consecutive days, positioned at the hip except when bathing or swimming. Participants were advised to continue their usual routine whilst wearing the device. Raw accelerometer data were extracted from the devices and re-integrated in 1-min epochs using ActiLife software™ (version 6) and processed using the package ‘accelerometry’ in R [19]. Validity of wear time was assessed using specifications described elsewhere [20]. Those with at least 4 days of wear time for 10 h/day were defined as having valid data. Cadence data were extracted and used without censoring. The following step activity measures were reported: Mean daily step count, mean step counts categorised according to the Adult Graduated Step Index: < 5000 steps (sedentary), 5000–7499 steps (low activity), 7500–9999 (somewhat active) and ≥ 10,000 (active) steps/ day [7], mean peak 1-min, 30-min and 60-min cadences in accordance with existing studies [16, 17] and the amount of time (minutes) and proportion of time (%) accumulated in previously defined cadence bands [18]: 0 (non-movement), 1–19 (incidental movement), 20–39 (sporadic movement), 40–59 (purposeful movement), 60–79 (slow walking), 80–99 (medium walking), ≥100 steps/minute (brisk walking or faster). Finally, to approximate whether recommended moderate intensity PA levels (i.e. 150 min/week or 30 min/day, 5 days/week) were being met through stepping activity the proportion achieving 30 min/day of stepping activity at brisk or faster walking pace (≥100 steps/minute) was calculated.

All analyses were conducted in STATA 14.2. Pearson’s pairwise correlation was performed to investigate the relationship between daily step count and peak 1-min, 30-min and 60-min cadence for the whole sample and by ethnic group. Assumptions were checked prior to calculating correlations. Descriptive statistics were calculated as frequencies (%) and mean (with standard deviation). Data is presented by the total population and by ethnic group (Chinese, Malay and Indian). Two-sample t-test and chi^2^ test were used to test differences between participant demographics as appropriate. Analysis of covariance and pairwise comparisons were performed to test for differences in continuous stepping parameters by ethnicity and differences in mean peak cadence by step categories. Multivariate logistic regression was used to test for associations between categorical stepping parameters and ethnic group. All analyses were adjusted for age, gender, marital status, education, diagnosis of arthritis, hypertension, diabetes or asthma, smoking status, alcohol drinking status, BMI and wear-time. A *p* value of < 0.05 was considered significant. Mean daily step counts were calculated for each participant by averaging the total steps/day across the days of wear, subsequently deriving the mean value. Mean peak cadences were calculated by ranking the individual cadence minutes and identifying the top 1 min for peak 1-min cadence and the mean of the top 30 and 60-min steps/minute (not necessarily consecutive minutes) for each participant. The mean peak 1-min, 30-min and 60-min cadence was then derived.

Approval was obtained from the National University of Singapore Institutional Review board. All participants provided written informed consent to take part in the study.

## Results

Out of the 895 participants who agreed to take part in the accelerometer measured PA study, 742 (83%) had valid accelerometer data. As per the inclusion criteria, 29 participants from other ethnic groups were removed leaving a total of 713 participants in the analyses. Table 1 presents the sample characteristics. Overall, 69% of participants were of Chinese, 17% of Malay and 14% of Indian ethnicity. Participants were mostly female (58%), with a mean age of 47.8 years. The majority were married, had moderate to high education levels and were employed. Gender, employment status, prior diagnosis of hypertension or arthritis did not significantly differ between ethnic groups. Malays in this cohort were significantly younger (compared to Chinese), were less likely to be married or drink alcohol and more likely to be smokers. A statistically significantly greater proportion of Malays had a lower level of education and lower monthly income compared to other ethnic groups. Those of Indian ethnicity were significantly more likely to be overweight and diabetic than Malay or Chinese. Those taking part in the SHS2 and the accelerometer measured PA study were not statistically different in age (45.9 v 47.8 years), gender (55% v 58% female), ethnicity (both 66% Chinese) respectively, marital status (64% v 61% married) or employment status (74% v 77% working), however education level was statistically higher in the accelerometer measured PA group.

**Table 1:** Sample characteristics

Participant characteristics	Total Sample *N* = 713^a^	Chinese *N* = 492 (69%)	Malay *N* = 121 (17%)	Indian *N* = 100 (14%)	*p* value
Mean age (SD)	47.8 (14.6)	49.1 (14.2)	43.9 (15.8)	46.5 (14.4)	*
Gender: Male (%)	296 (42%)	211 (43%)	44 (36%)	41 (41%)	0.42
Married (%)	426 (60%)	307 (62%)	53 (44%)	66 (66%)	< 0.001
Educational Level:					< 0.001
Low	167 (23%)	118 (24%)	30 (25%)	19 (19%)	
Medium	220 (31%)	135 (27%)	60 (49%)	25 (25%)	
High	326 (46%)	239 (49%)	31 (26%)	56 (56%)	
Working (%) *n* = 712	548 (77%)	380 (77%)	92 (76%)	76 (76%)	0.92
Monthly Income: *n* = 657					< 0.001
< S$2000	152 (23%)	100 (22%)	32 (28%)	20 (21%)	
S$2000–3999	194 (30%)	112 (25%)	51 (43%)	31 (32%)	
S$4000–5999	139 (21%)	94 (21%)	23 (20%)	22 (23%)	
S$6000+	172 (26%)	140 (31%)	9 (8%)	23 (24%)	
BMI: *n* = 712					< 0.001
Healthy < 23 kg/m^2^	298 (42%)	243 (49%)	35 (29%)	20 (20%)	
Overweight 23–27.4 kg/m^2^	280 (39%)	180 (37%)	48 (40%)	52 (52%)	
Obese ≥27.5 kg/m^2^	134 (19%)	68 (14%)	38 (31%)	28 (28%)	
Prior Diagnosis Of:					
Hypertension (%) *n* = 706	117 (17%)	80 (16%)	21 (18%)	16 (16%)	0.90
Diabetes (%) *n* = 706	56 (8%)	30 (6%)	11 (9%)	15 (15%)	0.01
Asthma (%)	72 (10%)	38 (8%)	18 (15%)	16 (16%)	0.007
Arthritis (%) *n* = 705	45 (6%)	28 (6%)	9 (8%)	8 (8%)	0.53
Current smoker (%)	98 (14%)	53 (11%)	29 (24%)	16 (16%)	0.001
Alcohol drinker (%)	345 (48%)	303 (62%)	6 (5%)	36 (36%)	< 0.001

^a^Total *n* = 713 unless stated otherwise. *BMI* Body Mass Index defined according to Singapore Health Promotion Board criteria, *SD* Standard Deviation. Education level: low (no formal qualifications/primary school leaving exam/secondary education), medium (0-levels/ A-levels), high (diploma/ university degree or equivalent). *t-test Age: Chinese versus Malay *p* < 0.001, Chinese versus Indian *p* = 0.09, Malay versus Indian *p* = 0.21

**Fig. 1:**
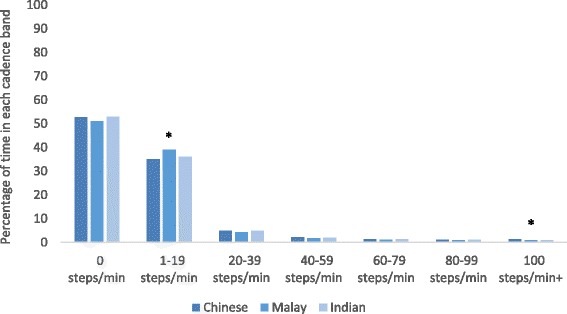
Average percentage of time spent in each cadence band by ethnicity. *Adjusted *p* < 0.05

The mean daily step count was 7549 steps and did not exceed 10,000 steps/day in any ethnic group (Table 2). Indians had a statistically significantly lower daily step count than Chinese participants, adjusting for baseline demographics (Indians 6888 steps versus Chinese 7614 steps *p* = 0.02). Other ethnic group comparisons did not reach statistically significant differences for daily step count. The largest step category across all ethnic groups (35–39% of participants) was 5000–7499 daily steps, which is classified as ‘low activity’ [7]. Malays had the highest proportion of participants achieving less than 5000 daily steps (26%) versus 23% of Indians and 14% of Chinese participants. Approximately half of accelerometer wear-time was spent at 0-steps/min (53%) and did not statistically differ across ethnic groups (*p* = 0.15, Table 2 and Fig. 1). Overall mean wear-time at >100steps/min (moderate intensity PA) was approximately 15 min and statistically significantly differences between the ethnic groups were apparent (adjusted *p* < 0.05). The overall proportion of participants achieving 30 min of moderate intensity PA through steps (≥100 steps/minute) was 27%. The proportion meeting this activity level was statistically significantly lower in Indian (15%) and Malay (16%) participants versus Chinese (32%) (unadjusted chi^2^ *p* < 0.001).

**Table 2:** Step activity by volume and intensity overall and by ethnic group

Stepping activity (all days)	Total population *N* = 713	Chinese *N* = 492	Malay *N* = 121	Indian *N* = 100	*p*-value
Mean daily wear time minutes (SD)	907.5 (116.8)	899.4 (112.5)	939.4 (131.5)	908.9 (112.9)	0.15
Mean daily steps (SD)	7549.8 (2847.1)	7745.2 (2774.4)	7140.6 (3073.3)	7083.9 (2839.0)	0.03
Daily steps by category (*n* and %):					
< 5000 steps/day	124 (17%)	70 (14%)	31 (26%)	23 (23%)	
5000–7499 steps/day	253 (36%)	171 (35%)	43 (36%)	39 (39%)	
7500–9999 steps/day	220 (31%)	169 (34%)	26 (21%)	25 (25%)	
10,000 or more steps/day	116 (16%)	82 (17%)	21 (17%)	13 (13%)	*
Mean daily wear-time minutes (SD) and % of time spent in each cadence band:					
0 steps/min (non-movement)	476.1 (126.7) 53%	475.6 (127.8) 53%	476.0 (135.0) 51%	478.7 (111.1) 53%	0.14
1–19 steps/min (incidental movement)	326.5 (89.7) 36%	316.1 (89.8) 35%	365.2 (84.2) 39%	330.6 (83.1) 36%	0.002
20–39 steps/min (sporadic movement)	47.4 (23.3) 5%	48.0 (23.4) 5%	46.1 (25.1) 4%	46.3 (20.9) 5%	0.34
40–59 steps/min (purposeful movement)	19.5 (10.3) 2%	19.7 (9.6) 2%	18.8 (12.9) 2%	19.0 (10.2) 2%	0.40
60–79 steps/min (slow walking)	12.2 (6.5) 1%	12.4 (6.1) 1%	11.5 (7.8) 1%	11.8 (6.9) 1%	0.24
80–99 steps/min (medium walking)	10.6 (6.8) 1%	10.9 (6.6) 1%	9.7 (6.8) 1%	10.7 (7.8) 1%	0.56
100–119 steps/min (brisk walking or faster)	12.2 (9.9) 1%	13.2 (9.9) 1%	10.4 (9.0) 1%	10.0 (10.1) 1%	0.04
≥120 steps/min (brisk walking or faster)	2.7 (4.6) < 1%	3.2 (5.1) < 1%	1.4 (3.0) < 1%	1.5 (3.6) < 1%	0.001
Mean peak 1-min cadence minutes (SD)	110.7 (17.3)	113.7 (15.7)	104.0 (19.5)	104.0 (18.0)	< 0.001
Mean peak 30-min cadence minutes (SD)	83.7 (21.4)	86.7 (20.2)	76.9 (22.8)	77.7 (22.3)	< 0.001
Mean peak 60-min cadence minutes (SD)	67.3 (20.3)	69.8 (19.4)	61.3 (21.2)	62.4 (20.9)	0.002
Proportion of participants accumulating 30-min/day at ≥100steps/minute (*n* and %)	190 (27%)	156 (32%)	19 (16%)	15 (15%)	**

*SD* Standard Deviation. *P*-values adjusted for age, gender, marital status, education, diagnosis of arthritis, hypertension, diabetes or asthma, smoking status, alcohol drinking status, BMI and wear-time. *Unadjusted chi^2^ *p* = 0.009, multivariate logistic regression Chinese ethnicity associated with < 5 k steps category (*p* = 0.04) and Chinese ethnicity associated with 7500–9999 step category (*p* = 0.009), all others non-significant. **Unadjusted chi^2^ *p* < 0.001, multivariate logistic regression Chinese odds ratio 2.6 *p* = 0.01, Malay odds ratio 0.4 *p* = 0.02, Indian odds ratio 0.5 *p* = 0.04

**Fig. 2:**
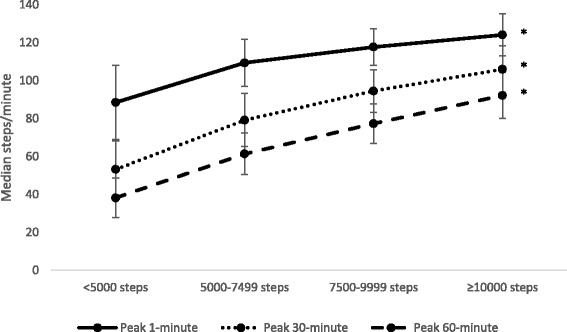
Mean (SD) peak 1-min, 30-min and 60-min cadence by step category. *Adjusted *p* < 0.001

Significant correlations were found between daily step count and peak cadences overall: 1-min (0.66), 30-min (0.79), 60-min (0.86) (steps/minute) and by ethnic groups: Chinese 0.62, 0.77, 0.84, Malay 0.68, 0.83, 0.89 and Indian participants 0.78, 0.84, 0.88, peak 1-min, 30-min and 60-min cadence respectively (all *p* < 0.001). Peak 1-min, 30-min and 60-min cadences are presented by stepping group categories in Fig. 2. The mean peak cadences increased significantly with stepping volume (*p* < 0.001).

## Discussion

This study contributes important insights into accelerometer measured PA in an urban Asian population. Stepping volume in this study was generally found to be low with a high degree of non-stepping time and low levels of moderate intensity stepping in all ethnic groups. This seems in line with research from the US which has shown limited contributions from stepping towards intensity based PA goals in adults [18]. Malays achieved the lowest stepping volume with 26% of them achieving less than 5000 steps/day, which has previously been indicated as an index of sedentary activity [7], compared to 23% of Indians and 14% of Chinese in this category. When exploring the lowest (0-steps: no step movement) and highest step intensity (≥100 steps/minute: moderate PA level) no significant differences between ethnic groups were observed for 0-steps/minute, however for ≥100 steps/minute ethnic group differences were statistically significant *p* < 0.05, although in all groups the amount of time was insufficient to meet PA recommendations.

Compared to other accelerometer studies the volume of PA from stepping was relatively low in our sample. A systematic review of pedometer recorded stepping activity in healthy adults reported average daily step counts ranging from 5003 to 13,800 (excluding one study in Amish) [21]. One of the largest accelerometer studies in 3725 Americans (the NHANES study) reported an uncensored mean daily step count of 9685 steps (confidence interval 9457–9912) [22]. The observed activity profile, specifically low volume, appears to be in line with self-reported research which reports urban Asian populations tend to do lower amounts of activity than Western populations [23]. Cadence information in free living populations is more limited. In comparison to NHANES study of 3522 participants stepping intensity was higher in our sample: mean peak 1-min cadence 110.7 versus 100.7 and peak 30-min cadence 83.7 versus 71.1 [16]. In other words, the population in this study appeared to walk shorter daily distances but walked at a faster pace compared to the American population. Yet, only a small proportion of time was spent contributing to moderate intensity PA: ~ 15 min at ≥100 steps/minute which is similar to the US study (~ 7 min) [18]. Furthermore, an extra 3 h/day was spent taking no steps (0-steps/minute) in our sample versus the US population: 7.9 h/day versus 4.8 h/day respectively [18]. This difference is not accounted for by differences in accelerometer wear-time validity criteria, with both this study and the NHANES study stipulating a minimum of 10 h daily wear-time. However, as the NHANES study does not report the mean wear-time it is feasible that some variation in wear-time may account for this difference.

In terms of the relationship between step volume and intensity significant correlations between the two dimensions of step activity were found in this study. Increasing step volume could therefore also result in meaningful improvements in intensity and thus meeting PA goals. However, in this population the mean peak 1-min and peak 30-min cadence only exceeded ≥100 steps/minute (moderate intensity PA) in those categorised in the 10,000 daily steps group and only 16% of the study population were in the 10,000 daily step group. The combination of limited PA levels and increased metabolic risk in a population of Asian descent is concerning [13]. One study estimated those of south Asian origin would need to accumulate 266 min/week of moderate intensity PA to exhibit a similar cardio-metabolic risk factor profile to European equivalents [24]. Achieving such high levels of activity seems infeasible given large proportions of global populations struggle to achieve 150 min/week, but this is not to say that lower levels of PA could not still benefit individuals. For example, a cohort study in 416,175 Taiwanese suggested that 15-min/day of moderate intensity PA could still reduce risk of mortality and increase life expectancy as compared with no moderate intensity PA [25]. Furthermore, a recent systematic review of cohort studies which investigated participation in non-vigorous PA concluded that the largest differences in mortality rate were observed between non-active and low activity groups, suggesting that avoiding inactivity alone is the most important step [26]. Others have proposed that the established 150 min/week PA goal may be unrealistic and may discourage participation in PA in those that are inactive [27] thus a reduced goal could be one strategy to improve population engagement in PA, but this approach would need to be validated. Instead promotion of some rather than no activity seems more appropriate than revision of global recommendations.

Other factors such as culture could also influence PA levels and what is perceived as PA. A previous investigation into self-reported PA in Singapore found that Malays were the most physically active with 79% meeting PA guidelines compared to 75% of Indians and 73% of Chinese [28], which is opposite to the accelerometer measured findings in this study. Differences between self-reported and accelerometer assessment of PA between ethnic groups have also been reported elsewhere. A UK study of white and south-Asian immigrants found no difference in accelerometer measured ambulatory behaviour but observed a much larger over-estimation of self-reported PA by white participants [29]. The differences in the perception of PA undertaken indicate a need to educate the population on PA and what contributes towards PA goals. Other factors such as socioeconomic status may also play a role. Education and income were lowest among Malay participants in our study and lower socio-economic status is known to be associated with less health conscious lifestyle behaviours [30]. Ethnicity and socioeconomic status have also been found to influence the types of PA undertaken. For example a cross-sectional study in Singapore reported that higher socio-economic status was associated with more leisure time activity and less household, occupational and transport activity [3, 31]. In sum, initiatives should consider the influence of ethnicity on PA perception and behaviours to improve the success of health promotion strategies.

This study investigated stepping patterns in a mixed ethnic population using an accelerometer. Other activities such as cycling, swimming and occupational activities which do not require stepping are not accurately captured by the accelerometer and thus some participants may be undertaking more PA than is reflected by the stepping activity reported. In addition, only a proportion of those who took part in the Singapore Health Study survey were included in this accelerometer based study. This may limit the generalisability of the study findings to the wider Asian population in Singapore and beyond as, for example, only those interested in PA may have taken part.

## Conclusion

Promotion of walking activity is an important strategy for achieving PA recommendations. This study suggests a need to improve walking volumes and intensity which by logic will also reduce large accumulations of non-movement. Future strategies should consider tailoring health promotion strategies by ethnicity to engage the population more broadly.

## Abbreviations

BMI: Body mass index
MET: Metabolic equivalent
NCD: Non communicable disease
PA: Physical activity
SD: Standard deviation
SHS: Singapore health study
WHO: World Health Organisation
